# Low Endotoxin Recovery—Masking of Naturally Occurring Endotoxin

**DOI:** 10.3390/ijms20040838

**Published:** 2019-02-15

**Authors:** Johannes Reich, Felix Alexander Weyer, Hiroshi Tamura, Isao Nagaoka, Hubert Motschmann

**Affiliations:** 1 Microcoat Biotechnologie GmbH, Am Neuland 3, 82347 Bernried am Stanberger See, Germany; f.weyer@microcoat.de; 2 LPS (Laboratory Program Support) Consulting Office, Tokyo 160-0023, Japan; htamura@lpsct.com; 3 Department of Host Defense and Biochemical Research, Juntendo University Graduate School of Medicine, Tokyo 113-8431, Japan; nagaokai@juntendo.ac.jp; 4 Institute of Physical and Theoretical Chemistry, University of Regensburg, 93040 Regensburg, Germany; Hubert.Motschmann@chemie.uni-regensburg.de

**Keywords:** low endotoxin recovery, endotoxin, lipopolysaccharide, limulus amoebocyte lysate, naturally occurring endotoxin, bacterial endotoxin testing, masking

## Abstract

Endotoxins are cell wall components of Gram-negative bacteria. A release of endotoxins into the human blood stream results in an inflammation reaction that can lead to life-threatening conditions like sepsis. Therefore, control for endotoxin contamination of intravenously administered drugs is crucial. Drugs are usually tested for putative endotoxin contamination with *Limulus*-based tests. However, validity of the compendial test procedures is questioned in the case of low endotoxin recovery (LER). To assure validity, regulatory authorities request hold-time studies of endotoxin in addition to pharmacopoeial requirements. Within these studies, endotoxin is added (spiked) to an undiluted product. The spiked product is held for a certain period of time and subsequently diluted for endotoxin determination. Due to the known heterogeneity of endotoxin the question has been raised as to which source represents the most adequate endotoxin spike. In the present study, endotoxin hold-time studies were analyzed by using different sources of endotoxin. Highly purified endotoxin, crude endotoxin extracts (Naturally Occurring Endotoxin) from different bacterial species and varied growth conditions as well as endogenous endotoxin contaminations were investigated. The results clearly demonstrate that endotoxin masking—an effect of LER—is dependent on the endotoxin source used. Various parameters such as bacterial strain and growth conditions lead to different masking susceptibilities. Due to these effects it is impossible to predict the susceptibility of bacterial endotoxin contamination to LER. In order to determine whether a sample is prone to LER, an endotoxin spike that is susceptible to LER is required.

## 1. Introduction

Endotoxins play an important role in the pathogenesis and manifestation of Gram-negative infections and in particular of septic shock. Lipopolysaccharides (LPS) are the dominating constituents of the outer leaflet of the outer membrane of Gram-negative bacteria ([1]). Therefore, the terms endotoxin and LPS are used interchangeably ([1,2,3,4]). Because the structures of LPS can vary significantly in its O-antigen, core region and lipid A, not all endotoxins possess the same toxicity [5]. In order to detect endotoxins, diverse detection methods like the rabbit pyrogen test [6,7], the monocyte activation test ([8,9,10], and the *Limulus*-based tests methods [11,12,13,14] are used. These methods have been used for decades in the pharmaceutical industry to test for potential contaminations of drug products with bacterial endotoxins. Due to their simplicity and high sensitivity [15,16], the *Limulus*-based tests are the most commonly used methods [15,16]. However, the effectiveness of the current test procedures is under debate regarding Low Endotoxin Recovery (LER). In 2013, Chen and Vinther presented LER, a masking effect of endotoxin in a matrix containing a chelator and a surfactant [17]. They showed that endotoxin spiked into an undiluted sample could not be recovered by dilution or by magnesium replacement, two methods commonly used to overcome test inhibition. After public recognition of the LER phenomenon, some publicized [4,18,19,20,21,22], and many not publicly accessible endotoxin recovery studies of biopharmaceutical drug products and their formulations were performed by endotoxin test providers, contract labs, and pharmaceutical companies. LER is defined as the inability to recover more than 50% activity over time when endotoxin is added to an undiluted product. LER cannot be overcome by dilution. LER and the associated masking effects have been widely confirmed, especially when tested drug products contained surfactants and chelators. Hold-time experiments are usually performed using standardized endotoxins such as the reference standard endotoxin (RSE) or the control standard endotoxin (CSE) as spike. However, since bacterial endotoxin tests are commercially used, the source and preparations of appropriate standard endotoxin are under debate [16]. The primary standard in bacterial endotoxin testing (BET) is RSE, which is endotoxin from *E. coli* O113:H10:K negative. RSE is purified in several steps including hot phenol-extraction [23]. For better handling, lactose and polyethylene glycol are added. This standard is accepted worldwide and sets the baseline for secondary standards. Because of limited RSE-availability, vendors of *Limulus*-based test systems distribute secondary standards which are calibrated against RSE. The preparations of such secondary standards are inspired by RSE, but the source can differ from *E. coli* O113. Exact production processes and formulations are not published. Since LER hold-time studies are widely performed, the source of the endotoxin is controversially discussed. It is especially debated, whether LER is dependent on the source of endotoxin [18,19,22,24,25]. Obviously, depending on source, preparation, and degree of purification, the LPS itself and the accompanying matrix components can vary respectively [26,27]. For instance, endotoxins from different bacteria can differ in their molecular structures [28,29]. There are differences in the lipid A (e.g., acylation), core region (e.g., substitution of sugar units) and O-antigen (e.g., distribution of sugar units). Moreover, depending on the preparation, endotoxin suspensions may vary in their compositions. For example, crude endotoxin extracts of bacteria, also known as Naturally Occurring Endotoxin (NOE), may contain substantial amounts of lipids and proteins, whereas phenol-extracted endotoxin preparations may only contain limited amounts of hydrophobic matrix components. Some experiments have proposed that the detectability of selected endotoxins in complex samples might be more robust compared to the detectability of commercially available standard endotoxins [18,19,30]. Therefore, endotoxins from different bacteria, grown under manipulated conditions, crude and highly purified endotoxins, as well as endogenous endotoxins were analyzed with regard to their detectability in a polysorbate/citrate matrix in the present study.

## 2. Results

### 2.1. Preparation and Characterization of Naturally Occurring Endotoxin (NOE)

In order to study the masking susceptibility of endotoxins from different bacteria, crude suspensions of bacterial endotoxin (NOE) were prepared from *E. coli, E. cloacae, S. marcescens, P. aeruginosa*, *B. cepacia*, *S. maltophilia* and *R. pickettii*. All bacteria were grown under equal, controlled, and defined conditions (See material and methods “prep a” for details). After 18 h of growth, the absorbance at OD (optical density) = 600 nm of all bacterial suspensions was determined as growth control (Table 1). While the bacterial culture of *E. coli* and *E. cloacae* showed the highest absorbance values at 600 nm (>1.7), lower absorbance values were obtained for *S. maltophilia* and *B. cepacia* (<0.7) (Table 1). This result indicating varying growth characteristics of different bacteria under fixed growth conditions.

Endotoxins are usually incorporated into the bacterial cell wall. During active proliferation, however, substantial amounts of endotoxins are released into the growth media via exocytosis. Thereby outer membrane vesicles (OMV), LPS monomers or fragments of the bacterial outer membrane resulting from disrupted bacterial cells can be released. All of these particles, besides the actual bacteria, contribute to the endotoxicity (detectable endotoxin activity with bacterial endotoxin test) of the sample. In order to prepare sterile endotoxin spike material, sterile-filtrated supernatants of bacterial suspensions were used and endotoxin activity determined. As a consequence, bacterial cells and bigger breakdown products are removed and their activity does not contribute under given conditions. The measured endotoxin activity of the filtrate of different bacteria suspensions spanned a broad range from approximately 400 to 400,000 EU/mL, although equal growth conditions were used for all stains. Only a weak correlation between the measured OD 600 and endotoxin activity values was observed.

The endotoxicity of different species varies, indicating that endotoxicity seems to be species-dependent.

**Figure 1:**
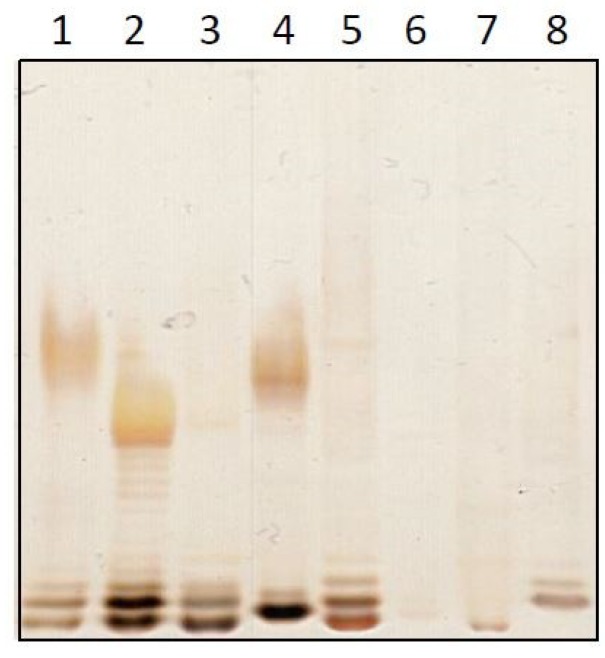
Analysis of crude bacterial extracts. A sodium dodecyl sulfate polyacrylamide gel electrophoresis (SDS-PAGE) of the crude extracts was silver-stained. The lanes reflect endotoxins from *E. coli* O55:B5 (1), *E. coli* O113 (2), *E. cloacae* (3), *S. marcescens* (4), *P. aeruginosa* (5), *B. cepacia* (6), *S. maltophilia* (7) and *R. pickettii* (8).

Endotoxins were applied to sodium dodecyl sulfate polyacrylamide gel electrophoresis (SDS-PAGE) and detected by silver staining (Figure 1). The typical ladder pattern of LPS could be observed in most lanes. The varying intensities of the bands reflect different concentrations, which were approximately in agreement with the detected activities (Figure 1 and Table 1). Upon closer examination, variations in the arrangement of bands between the different endotoxin samples could be observed.

These reflect different molecular structures and again confirm the heterogeneity of endotoxins. Taken together, crude preparations of endotoxin from different species grown at the same growth conditions show a variable endotoxicity and are heterogeneous in their structure.

### 2.2. Analysis of the Masking Ability of Different NOE

**Figure 2:**
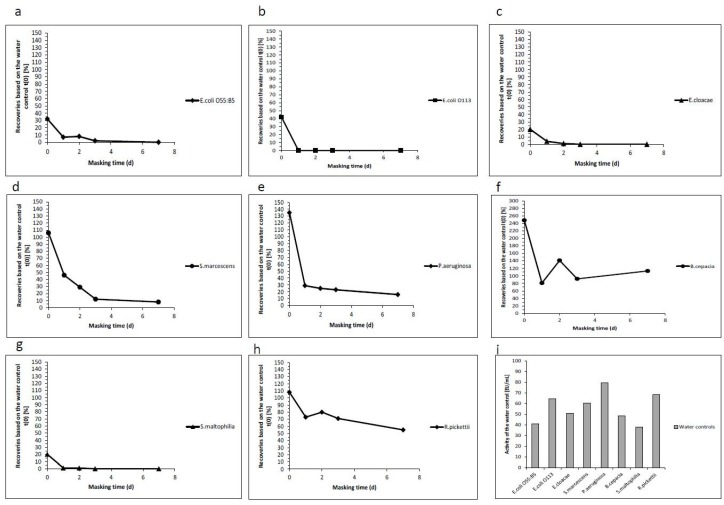
Hold time study using NOEs of different species. Endotoxin recovery over seven days at room temperature are shown for different species (*E. coli* O55:B5 (**a**), *E. coli* O113 (**b**), *E. cloacae* (**c**), *S. marcescens* (**d**), *P. aeruginosa* (**e**), *B. cepacia* (**f**), *S. maltophilia* (**g**) and *R. pickettii* (**h**)). For spiking, a 10-fold concentrated stock solution was prepared. The concentration of the water controls corresponding to the activities (EU/mL) are shown in (**i**). For the recovery experiments, the crude extracts were spiked into 0.05% (w/v) polysorbate 20 and 10 mM sodium citrate. The recoveries were determined after 0, 1, 2, 5 and 7 days. All recoveries were calculated based on the corresponding water control at time point 0.

Recent publications have questioned the LER phenomenon. They showed that a “laboratory-derived endotoxin from *E. coli*” was not masked after spiking it into three different batches of a monoclonal antibody formulated in a polysorbate 80/citrate buffer [18]. Similar results were obtained using a Natural Occurring Endotoxin from *Enterobacter cloacae* [25,31]. To challenge these observations, NOEs derived from eight different bacterial strains (*E. coli O55:B5, E. coli O113, E. cloacae, S. marcescens, P. aeruginosa, B. cepacia, S. maltophilia* and *R. pickettii*), grown under similar conditions, as described in the chapter above, were used as an endotoxin spike for masking experiments in a polysorbate 20/citrate matrix (Table 2 and Figure 2). Crude extracts of endotoxin from *E. coli*, *E. cloacae* and *S. maltophilia* resulted in low recovery already at day 0. In contrast, endotoxin from *S. marcescens*, *P. aeruginosa*, and *R. pickettii* showed a gradual loss of recovery over time. The endotoxin from *B. cepacia* could be detected over time and showed no trend in reduced activity.

These results show, in clear contrast to the aforementioned data, that NOEs from different bacteria, but grown and prepared under equivalent conditions, exhibit different masking susceptibilities and can be masked in a polysorbate 20/citrate matrix.

### 2.3. Analysis of the Masking Ability of Different NOEs Depending on Their Growth Conditions

**Figure 3:**
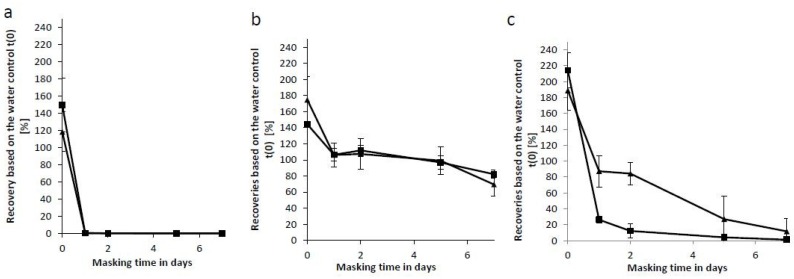
Masking ability of different NOEs depending on the growth condition. Endotoxin recovery was plotted as function of incubation time. 100 EU/mL endotoxin from different bacteria ((**a**) *E. coli* O113:H21, (**b**) *B. cepacia* and (**c**) *P. aeruginosa*) were spiked into samples containing 10 mM sodium citrate and 0.05% (w/v) polysorbate 20 and incubated at room temperature (RT). The used endotoxin extracts were derived from two different bacterial growth conditions. Squares reflect recovery of endotoxin from bacteria grown at 37 °C using 100% LB media. Triangles reflect recovery of endotoxin from bacteria grown at room temperature using 1% (v/v) LB media. Each endotoxin was prepared in triplicate. The corresponding endotoxin measurements of three repetitions were analyzed on the same microtiter plate. For calculation of the data points the mean value of the three individual preparations was used. The error bars represent the standard deviation of the three replicates.

Every preparation of LPS is a heterologous mixture of various LPS molecules [32,33]. While all LPS molecules contain the lipid A moiety and polysaccharides, the degree of acylation, substituents (e.g., aminoethanol) as well as the sugars (e.g., O-antigen) varies. Additionally, bacteria might adapt to the environmental growth conditions, which results in modifications of the LPS molecule. One potential change due to environmental conditions is the modification of an LPS-phosphate group with an amino-arabinose group [34]. In order to exclude that the observed masking effects of NOEs in a polysorbate 20/citrate matrix were caused by growth conditions, we performed a similar experiment with endotoxin derived from two different growth conditions. Three bacterial strains, *E. coli O113*, *P. aeruginosa* and *B. cepacia* were grown under conditions including either rich-nutrition media (100% LB) at elevated temperatures (37 °C) or low-nutrition (1% LB) at room temperatures (prep b). NOEs were prepared as described before. A buffer containing 10 mM sodium citrate and 0.05% (w/v) polysorbate 20 was spiked with 100 EU/mL from both preparations and held for up to 7 days, respectively (Figure 3a). The endotoxin from *E. coli* O113 was not recovered independent of growth conditions after one day of masking. In contrast, the recoveries of crude extract from *B. capecia* showed no significant decline (Recovery >50%), independent of growth condition (Figure 3b). Recovery of endotoxin from *P. aeruginosa* depend on growth conditions (Figure 3c). For both *P. aeruginosa* preparations the same recovery trends are observed, but the recovery dropped below 50% already within one day (bacteria grown at 37 °C using 100% LB media). For the second preparation, the recovery dropped below 50% only after 2 days. Thus, modified growth conditions resulted in diverging masking kinetics.

Taken together, this result demonstrates that masking can be dependent on the growth conditions and the nutrition content in the media.

### 2.4. Comparison of the Masking Behavior of Purified Lipopolysaccharides (LPS) and NOE

**Figure 4:**
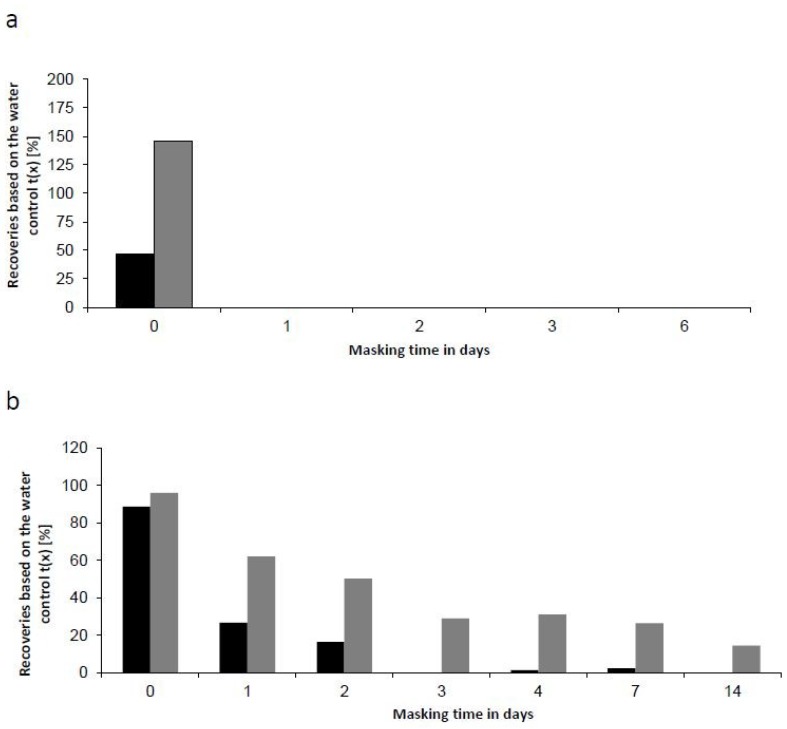
Differences in the masking behavior of purified endotoxins and NOEs. Recovery of endotoxin from *E. coli* O55:B5 is shown over incubation time. 100 EU/mL of gel-filtrated endotoxin (black bars) and sterile filtrated bacterial suspension (grey bars) were spiked into samples containing 10 mM sodium citrate and 0.05% (w/v) Polysorbate 20. The samples were incubated up to 14 days at RT (**a**) and 4 °C (**b**).

For this study we analyzed NOEs from different species. However, in the pharmaceutical industry purified LPS preparations are used as reference standard endotoxin and as control standard endotoxin. These standards are purified by phenol-chloroform extraction and further steps (e.g., size exclusion chromatography). They are the endotoxin of choice when masking is analyzed in hold-time studies. As most of the studies that are used to analyze masking and its mechanism are performed using purified LPS preparations [20,21,35], masking for purified LPS preparations is well established. Purification of spike material is the basis for standardization because of its required identity and known purity [36]. To further evaluate the impact of endotoxin purification, endotoxins from one bacterial species (*E. coli* O55:B5) were prepared by different methods and subsequently analyzed. Phenol-extracted endotoxin and crude supernatants of bacterial suspension were spiked into a polysorbate 20/citrate matrix and incubated for up to six days at room temperature (Figure 4a). Both preparations showed no recovery after one day of incubation, which confirms the pronounced masking susceptibility of endotoxin from *E. coli*.

Due to the fast kinetics of masking, the experiment was also performed at decreased incubation temperature 2 to 8 °C (Figure 4b). The reduced incubation temperature was chosen, as endotoxin masking can be decelerated, allowing a better resolution of slight differences in masking susceptibilities (Figure 4b). After 2 days of incubation, 50% and 14% after 14 days of the initial endotoxin content could be detected within the NOE at 2 °C to 8 °C. In comparison, the recovery of phenol-extracted endotoxin was already low after one day of incubation (27%) and no significant endotoxin content was detectable after three days of incubation. At lower incubation temperature the detectability of NOE decreased slower, compared to phenol-extracted endotoxin. However, both preparations of endotoxin were affected by masking.

In consequence, endotoxin-masking kinetics can be affected by the extraction method of endotoxin, but the kinetics rather depends on the source of bacteria than on the purification state of the endotoxin.

### 2.5. Masking Behavior of an Endogenous Endotoxin Contamination

**Figure 5:**
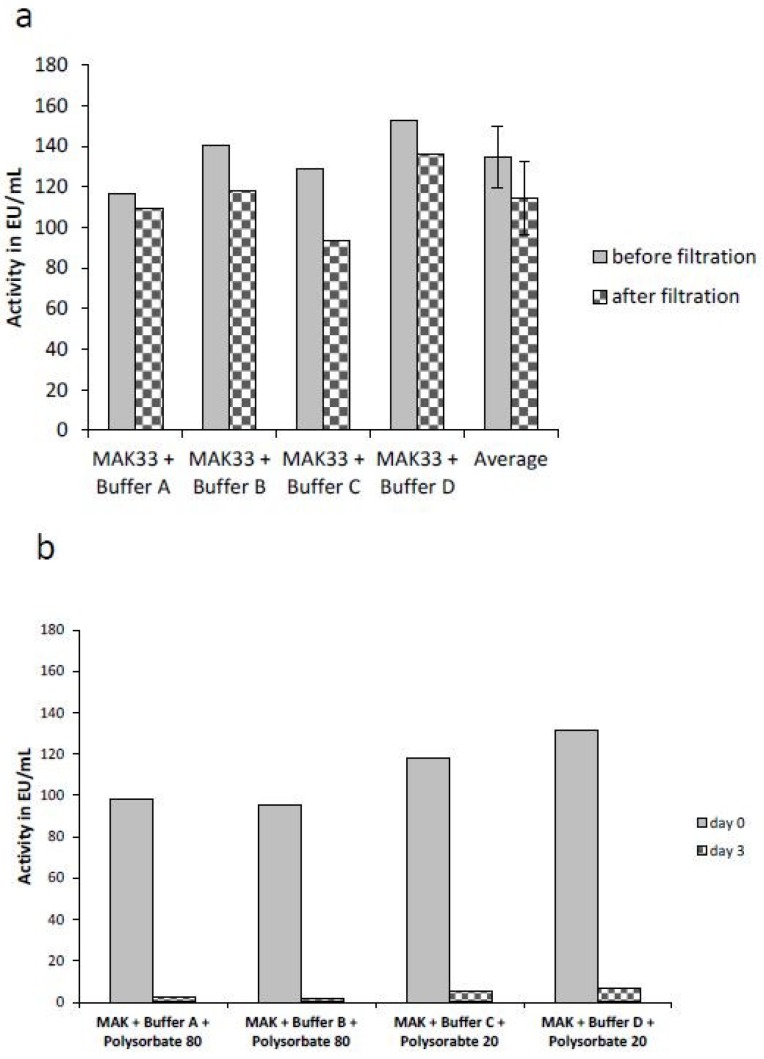
Masking of an endogenous endotoxin contamination. Endotoxin activity of an unknown endotoxin contamination under different buffer conditions is shown. 10 mg/mL monoclonal antibody (MAK33) were solubilized in 25 mM sodium citrate, pH 6.5 (buffer A); 10 mM sodium citrate, pH 7.5 (buffer B); 160 mM trehalose, 50 mM sodium phosphate pH 6.2 (buffer C) and 10 mM sodium phosphate, pH 7.5(buffer D). Endotoxin content was determined before and after sterile filtration (0.2 µm) (**a**). For endotoxin detection EndoZyme^®^ was used. After filtration 0.07% (w/v) and 0.05% (w/v) polysorbate 80 were added to the sample in buffer A and B, respectively. 0.04% (w/v) and 0.05% (w/v) polysorbate 20 were added to the sample in buffer C and buffer D, respectively. Endotoxin activity was determined immediately after addition of polysorbate (day 0) and after incubation of three days (day 3) at RT (**b**). For endotoxin detection the EndoLISA^®^ was used.

For all of the examples above, the source of endotoxin was known. Endotoxins were consciously added to samples containing surfactants and chelators. In order to examine a real endotoxin contamination, the detectability of an endogenous contaminated monoclonal antibody was analyzed. The lyophilized antibody was solubilized in four different chelator-containing buffer systems (A: 25 mM sodium citrate pH 6.5, B: 10 mM sodium citrate pH 7.5, C: 160 mM trehalose and 10 mM sodium phosphate pH 6.2 and D: 10 mM sodium phosphate pH 7.5). An average endotoxin activity of 135 EU/mL and 114 EU/mL was determined before and after sterile filtration, respectively (Figure 5a). The different buffer systems as well as the filtration had no major effects on the detectability of the endogenous endotoxin contamination of the antibody. Only small differences between the activity before and after the filtration step were observed. As expected, no masking was observed in the buffer systems containing only the chelating agent. As is known from previous studies on the mechanism of LER [20,21], masking is driven by a simultaneous presence of a surfactant (e.g., Polysorbate 20 or Polysorbate 80) and a chelator (e.g., sodium citrate). Therefore, 0.07% (w/v) of the surfactant Polysorbate 80 was added to the antibody in buffer A, 0.05% (w/v) Polysorbate 80 to the antibody in buffer B, 0.04% (w/v) Polysorbate 20 to the antibody in buffer C and 0.05% (w/v) Polysorbate 20 to the antibody in buffer D, respectively. The endotoxin concentration was directly determined after adding the detergents (day 0). Comparable activities as determined directly after filtration were observed (Figure 5a). After an incubation of 3 days at room temperature, the endotoxin content was determined again. A significant decrease (90% to 98%) of the endotoxin activity could be observed in all four samples (Figure 5b).

Endotoxin masking was observed in all four antibody preparations, independent of the composition of the masking buffer. According to previous publications, all of these buffers have masking capabilities [20,21]. Thus, the results are consistent with the above mentioned data and literature, no further replicates or statistical analysis of the data were performed. The present data show a series of experiments, in which NOEs can be masked under LER-conditions, but with various masking kinetics. Individual masking kinetics are consistent when reproduced (Figure 3) or compared to other experiments (Figure 2, Figure 3 and Figure 4). Finally, the data clearly demonstrates that an endogenous endotoxin contamination, which reflects a real naturally occurring endotoxin, is masked in a commonly occurring formulation buffer of biologicals.

## 3. Discussion

Endotoxins are complex molecules originating from the outer membrane of Gram-negative bacteria containing a hydrophobic lipid moiety and a polysaccharide moiety. The polysaccharide part of the endotoxin is highly diverse and varies in number of sugars within a unit, the nature of the linkages of the sugars, as well as the number of repetitive units. O-antigen sugars appear to be most variable, core structures appear to be less variable. In contrast, lipid moieties of LPS are considered highly conserved within a genus [1,37]. Gram-negative bacteria exhibit only minimal growth requirements and can thus be found in clean water, processed water, or buffers [38], required for the production of biopharmaceuticals. As endotoxins are strong stimulators of the human immune system mediated by the Toll-like receptor 4 complex, monitoring of endotoxins is a crucial step in the production of biopharmaceuticals. While the *Limulus*-based tests are the method of choice to test for a potential endotoxin contamination, these have been challenged under the light of the low endotoxin recovery phenomenon. To evaluate whether a sample is affected by LER, regulatory authorities request hold-time studies to evaluate detection of an endotoxin spike in an undiluted drug product over time, but the kind of endotoxin used as spike was under debate. Recent studies suggested the use of NOE as standards for hold-time studies instead of purified endotoxin. Moreover, it is hypothesized that masking of endotoxin is mainly induced by the use of purified endotoxin [39,40]. The authors claim that the use of NOEs is closer to contaminations observed during biopharmaceutical drug production. Purified endotoxins are not observed in nature and exhibit different properties due to the extraction process and missing pieces like proteins or lipids. Hence, to investigate whether the phenomenon of LER is limited to standardized endotoxins from *E. coli*, detectability of NOEs from different bacterial species was studied in a typical biopharmaceutical drug product matrix containing polysorbate and sodium citrate. Such a matrix has been shown to induce LER. In this work, it could be shown that some NOEs were masked as well. However, masking of NOEs was shown to depend on species and growth conditions. While no masking was observed over a 7-day period for NOEs from *B. cepacia* and *R. pickettii*, masking was observed for crude extracts of *E. coli, E. cloacae, S. marcescens, P. aeruginosa* and *S. maltophilia*. These results show endotoxins which are rapidly affected by LER (e.g., *E. coli* O113) and endotoxins which are less affected by LER. Endotoxin from *B. cepacia* was not affected by masking under given conditions. Regarding *B. cepacia*, it is described in the literature that the LPS possess an unusual structure. The bacteria lower the anionic charge of the cell surface by the substitution of 4-amino-4-deoxyarabinose residues bound to phosphates of the lipid A backbone [41,42,43]. This might explain the observed limited masking susceptibility. These results would prove NOEs unsuitable for endotoxin hold-time studies. The absence of masking in a drug product could be due to the analyzed species and its respective growth conditions. However, masking would be observed in this exact drug product, if a different species had been used. It has to be pointed out that not all endotoxins are similarly affected when growth conditions are modified. A change of masking susceptibility cannot automatically be observed when comparing recovery kinetics of endotoxins from the same source, but derived from different growth conditions.

These results can be explained with an adaptation of the bacteria to an unfavorable environment to ensure viability. It is known that bacteria are able to modify their primary LPS structure under certain growth conditions. Thereby they can reinforce the external membrane to assure optimal protection against the environment [34]. Recent publications have claimed that the endotoxin extraction method is the reason for LER, as the endotoxin is extracted from its native environment and can adopt non-natural aggregation states. The present findings, using highly purified endotoxin and NOE from the same species (*E. coli* O55:B5), show that masking is not caused by the purification process itself, as both preparations were similarly masked. At decreased temperature, the masking process of the NOE was slower than observed for the purified endotoxin. These results indicate a change in the masking kinetics, probably due to the additional elements like proteins and lipids within the crude extract. However, masking characteristics remained unchanged, independent of the purification process. Taken together, masking characteristics of a given endotoxin preparation are mainly influenced by growth conditions, not by the purification process. With the exception of Schwarz et al. [4], all published studies either used a purified endotoxin standard or a lab-derived NOE to analyze masking and to simulate a possible contamination. Within this study, the masking behavior of an endogenous endotoxin contamination affecting a commercial antibody was analyzed, as this represents a realistic case. The contamination is observed in the final product and the bacterial species and source of the contamination is unknown. This represents a scenario that pharmaceutical quality control departments face during product release. The masking behavior of this contamination was analyzed using four commonly used buffers for drug product formulation. Within all four buffers, rapid masking was observed in the tested product. Less than 10% of the initial endotoxin activity was measured after three days. These results clearly show that endotoxin masking is not linked to the purification process, but rather to the molecular structure. This emphasized that NOEs are no solution to overcome LER, as masking was also observed for an endogenous endotoxin contamination. The use of NOEs in hold-time studies could instead be misleading, as masking would not be observed with the analyzed product, but might indeed be observed with an endogenous contamination.

The origin of LPS is inevitably connected to the respective bacteria. However, an intrinsic heterogeneity is certain. It is impossible to predict source and cause of potential bacterial endotoxin contaminations of a sample. As a consequence, the masking susceptibility of a potential endotoxin contamination is unknown, too. In order to ensure reliable detection of endotoxins, the masking capability of a sample has to be evaluated. To analyze the masking capability of a sample, endotoxin hold-time studies have to be performed with endotoxin spikes, that are susceptible to masking. The results above have shown that standardized endotoxins from *E. coli* exhibit a pronounced susceptibility to endotoxin masking and represent an appropriate source for endotoxin hold-time studies. With regard to the heterogeneity of endotoxins and the diversity of sample compositions, a panel of different endotoxins might be the safest way to determine the masking capability of a sample and to ensure detectability of a potential contamination. A reference standard and an additional control standard from different species could be used as such a panel. Lastly, as soon as a sample is identified with the capability of endotoxin masking, a suitable detection method needs to be developed in order to properly detect endotoxins and to avoid the underestimation of a contamination.

## 4. Material and Methods

### 4.1. Chemicals and Materials

Polysorbate 20, polysorbate 80, sodium chloride, sodium azide, trisodium citrate, phosphoric acid, sodium dihydrogenphosphate, potasium dihydrogenphosphate, disodium hydrogen phosphate-heptahydrate, 2-amino-2-(hydroxymethyl)-1,3-propanediol (tris), 2-mercaptoethanol, isopropanol, d(+)-glucose, sodium chloride, calcium dichloride and magnesium dichloride were obtained from Sigma-Aldrich Chemie GmbH, Steinheim, Germany. Ammonium hydroxide, formaldehyde and d(+)-trehalose-dihydrate were obtained from AppliChem GmbH, Darmstadt, Germany. Acetic acid, glycerol, periodic acid, sodium hydroxide, silver nitrate, sodium dodecylsulfate (SDS) and yeast extract (powdered) were obtained from Carl Roth GmbH & Co. KG, Karlsruhe, Germany. Bromophenol blue sodium salt was obtained from Merck Chemicals GmbH, Darmstadt, Germany. 20× Tris-tricine/SDS electrophoresis buffer were obtained from Serva Electrophoresis GmbH, Heidelberg, Germany. The mouse monoclonal antibody (MAK33) was obtained from Roche Diagnostics Deutschland GmbH, Mannheim, Germany. Tryptone Bacto TM was obtained from Becton Dickinson GmbH, Heidelberg, Germany. Depyrogenated water, depyrogenated borosilicate glass tubes and recombinant Factor C tests, EndoZyme^®^ and EndoLISA^®^ were obtained from Hyglos GmbH, Bernried, Germany. Prior to the experiments, all relevant materials were tested for endotoxins and proven to contain less than 0.05 EU/mL.

### 4.2. Endotoxins and Bacteria

Endotoxin from *Escherichia coli* O55:B5 (gel-filtrate) was obtained from Sigma-Aldrich Chemie, Steinheim, Germany. Freeze-dried bacteria from *E. coli* O55:B5 (DSM 4779), *Enterobacter cloacae* (DSM 30054) and *Pseudomonas aeruginosa* (DSM 500 71) were obtained from Leibniz Institute DSMZ-German Collection of Microorganisms and Cell Cultures, Braunschweig, Germany. Freeze-dried bacteria from *E.coli* O113 (Ecor 30) was obtained from Escherichia coli Reference Collection, East Lansing, USA. Freeze-dried bacteria from *Burkholderia cepacia* (2008 B02-12.20.164) and *Stenotrophomonas maltophilia* (DSMZ 50 170) were obtained from Robert Koch-Institute, Wernigerode, Germany. Bacteria from *Ralstonia pickettii* (isolate) were a kind gift from Hyglos GmbH, Bernried, Germany.

### 4.3. Preparation of Crude Endotoxin Extracts (Naturally Occurring Endotoxins, NOEs) (Prep a)

For bacterial growth, 5 mL lysogeny broth (LB) media (10 g/L sodium chloride, 5 g/L yeast extract and 10 g/L tryptone) were inoculated with the desired bacterial strain, followed by incubation overnight at 37 °C in a shaking incubator (Platform shaker: Innova 2300, New Brunswick Scientific Co, Enfield, USA; Incubator: Wärmeschrank für Plattformschüttler, Mytron Bio- und Solartechnik GmbH, Heilbad Heiligenstadt, Germany). Afterwards, 200 µL of the preparatory culture were transferred into 500 mL of media (12.8 g/L disodium hydrogenphosphat-heptahydrat, 3 g/L potassium dihydrogenphosphat, 0.5 g/L sodium chlorid, 1 g/L ammonium chloride, 0.01 g/L calcium dichloride and 0.4 wt% glucose) and incubated for 24 h at 37 °C. Growth of bacteria was monitored by light absorption at 600 nm using a spectro photometer (V550 Jasco Germany GmbH, Gross-Umstadt, Germany). Bacterial growth was stopped by temperature reduction to 4 °C. Crude bacterial extracts (Natural occurring endotoxins) were prepared by centrifugation of the culture at 4500 rpm (Heraeus Multifuge 3 S-R) and sterile filtration (0.2 µm, Pall GmbH, Dreieich, Germany) of the supernatant. For conservation 0.05% (v/v) sodium azide were added. Required endotoxin concentrations for endotoxin recovery studies were adjusted by dilution with depyrogenated water.

### 4.4. Preparation of NOEs from Bacteria Grown under Low-Nutrition Conditions (Prep b)

Five mL LB media (10 g/L sodium chloride, 5 g/L yeast extract and 10 g/L tryptone) were inoculated with the desired bacterial strain, followed by incubation overnight at 37 °C in a shaking incubator (Platform shaker: Innova 2300, New Brunswick Scientific Co, Enfield, USA; Incubator: Wärmeschrank für Plattformschüttler, Mytron Bio- und Solartechnik GmbH, Heilbad Heiligenstadt, Germany). Afterwards, the preparatory culture was transferred into 20 mL of LB media or a minimal media (1% LB media in depyrogenated LRW) and incubated for 18 h at room temperature. Bacterial growth was stopped by temperature reduction to 4 °C and sterile filtration (0.2 µm) of the bacterial suspension. For conservation 0.05% (v/v) sodium azide was added. Required endotoxin concentrations for endotoxin recovery studies were adjusted by dilution with depyrogenated water.

### 4.5. Sodium Dodecyl Sulfate Polyacrylamide Gel Electrophoresis (SDS-PAGE) and Silver Staining

The NOEs of all different species were vortexed for 30 s. 40 µL of each sample were mixed with 10 µL sodium dodecyl sulfate (SDS) loading buffer and boiled at 100 °C for 10 min. 18 µL of these samples were loaded on a 20% gradient Tris-Tricin gel (Anamed Elektrophorese GmbH, Rodau, Germany). The sodiumdodecylsulfatepolyacrylamid gelelectrophoresis was performed in Tris-Tricine/SDS buffer at 130 V for 90 min. The gel was fixed overnight in fixation solution (25% (v/v) isopropanol and 7% acetic acid) and oxidized for 5 min using an oxidation solution (0.06 g/mL periodic acid, 0.33% (v/v) Isopropanol and 0.09% (v/v) acetic acid. The gel was washed 5 times in depyrogenated water and silver stained for 10 min in staining solution (350 µL sodium hydroxide (8 M), 1 mL concentrated ammonium hydroxide (28% (w/v), 2 mL silver nitrate (20% w/v) and 75 mL depyrogenated water). The stained gel was washed 5 times with depyrogenated water and developed for 10 min with the development solution (1 mg/mL citric acid, 0.037% formaldehyde). The development was stopped using acetic acid (10% v/v).

### 4.6. Sample Masking Preparation for Masking Kinetics

Samples were prepared in 5 mL glass tubes with sample volumes of 1 mL per sample. Unless otherwise described, samples were spiked with a 10-fold or concentrated stock solution of the respective endotoxin. The pH of buffer components was adjusted to 7.5 if not otherwise described. Before adding the spikes to the sample, the stock solution was shaken at 1400 rpm for 10 min. After spiking, the samples were incubated at defined temperatures and for defined periods of time. Immediately after incubation, the samples were mixed at 1400 rpm for 2 min again, and diluted in depyrogenated water in order to avoid test interference. Necessary dilutions of the particular sample compositions were determined prior to the actual experiment.

Samples with different incubation periods were measured on the same microtiter plate, to avoid variation from test to test. Therefore, endotoxin recovery kinetics was performed in a reverse manner. The particular sample was aliquoted and all aliquots were stored under equal conditions over time. The aliquot with the longest endotoxin incubation period was spiked first (e.g., 7 days prior to the measurement). Further aliquots with shorter incubation periods were spiked later in accordance with the respective incubation period. The zero-time point aliquot was spiked immediately before measurement. Resulting kinetics consists of various time points and reflects one experiment, if not otherwise indicated.

The validity of the actual measurement was controlled by spiking the defined endotoxin amounts into the diluted samples (Positive Product Control (PPC)). Endotoxin determination in a diluted sample was considered valid, if 50% to 200% of the spiked endotoxin (PPC) was recovered. To control the accuracy of the endotoxin spiked into the undiluted samples, equal amounts of endotoxin were spiked into depyrogenated water (water control), mixed and identically incubated as the actual sample. The spike into the undiluted sample was considered valid if the water control was in the range of 50% to 200% of the theoretical expected spike concentration.

For calculation of the recovery, the determined endotoxin concentrations in the tested samples were compared to the endotoxin concentrations at time zero in water controls and stated as percent.

### 4.7. Sample Preparation using an Endogenous Endotoxin Contamination

The lyophilized monoclonal antibody (MAK33) was solubilized at a concentration of 10 mg/mL in buffer A (25 mM sodium citrate, pH 6.5), buffer B (10 mM sodium citrate, pH 7.5), buffer C (160 mM Trehalose, 50 mM sodium phosphate pH 6.2) and buffer D (10 mM sodium phosphate, pH 7.5). The endotoxin concentration of the natural contamination was determined before and after sterile filtration using the EndoZyme^®^ assay. After filtration, 0.07% (v/v) polysorbate 80 were added to the sample in buffer A, 0.05% (v/v) polysorbate 80 were added to the sample in buffer B, 0.04% (v/v) polysorbate 20 were added to the sample in buffer C and 0.05% (v/v) polysorbate 20 were added to the sample in buffer D, respectively. The endotoxin content was determined immediately after the addition of polysorbate (day 0) and after incubation at room temperature (19–25 °C) after 3 days (day 3) using the EndoLISA^®^ assay. For calculation of endotoxin recovery, the determined endotoxin concentrations in the actual sample was compared to the determined endotoxin concentration in the water control stated as percentage.

### 4.8. Endotoxin Detection

For bacterial endotoxin detection, Limulus-based tests (rFC) were used according to manufacturer’s instructions. The amount of fluorescence substrate (amino-methylcoumarin) released was measured fluorometrically at 440 nm (Excitation: 380 nm) with a FLx800 fluorescence microplate reader (BioTek Instruments GmbH, Bad Friedrichshall, Germany). All samples were measured in duplicate and average values were used for further calculations. Standard curves were fitted using a four-parameter logistic non-linear regression model. The detection limit of the assay was 0.005 EU/mL (EndoZyme^®^) and 0.05 EU/mL (EndoLISA^®^). If not otherwise indicated, EndoZyme was used for endotoxin detection.

Microsoft Excel 2010, Version 14.0.7015.1000 was used to calculate endotoxin recovery, plot graphs and to simulate endotoxin recovery kinetics. Gen5 Data Analysis Software Version 2.05 from BioTek Instruments GmbH, Bad Friedrichshall, Germany was used to calculate standard curves for determination of endotoxin concentrations.

## Figures and Tables

**Table 1:** Correlation of bacterial growth behavior and endotoxin activity. To monitor the growth of different Gram negative bacterial species (*E. coli O55:B5*, *E. coli O113*, *E. cloacae*, *S. marcescen*, *P. aeruginosa*, *B. cepacia*, *S. maltophilia* and *R. pickettii*), the OD 600 was determined before harvesting and correlated with the activity of the prepared crude extract.

Source	Absorbance	Activity
	(600 nm)	(EU/mL)
*E. coli O55:B5*	1.9	146,174
*E. coli O113*	1.7	402,789
*E. cloacae*	1.7	189,103
*S. marcescens*	1.4	116,175
*P. aeruginosa*	0.9	8595
*B. cepacia*	0.7	357
*S. maltophilia*	0.3	4557
*R. pickettii*	0.8	77,815

**Table 2:** Hold time study using naturally occurring endotoxins (NOEs) from different species. The hold time study was performed over 7 days using the prepared NOEs for different species. All recoveries in percentage were calculated based on the water control at *t*(0).

Masking Time in Days	Endotoxin Recoveries Based on the Water Control *t*(0) (%)					Activity of the Water Control (EU/mL)
	0	1	2	3	7	
*E. coli O55:B5*	32	7	8	2	0	40.9
*E. coli O113*	42	0	0	0	0	64.7
*E. cloacae*	20	4	1	0	0	50.9
*S. marcescens*	106	46	29	12	8	60.5
*P. aeruginosa*	135	29	25	23	16	79.6
*B. cepacia*	248	81	141	92	113	48.5
*S. maltophilia*	20	1	1	0	0	37.9
*R. pickettii*	108	73	80	71	55	68.4
